# *Echinococcus multilocularis* (Cestoda, Cyclophyllidea, Taeniidae): functional ultrastructure of the penetration glands and nerve cells within the oncosphere

**DOI:** 10.1007/s00436-018-5957-9

**Published:** 2018-06-09

**Authors:** Zdzisław Świderski, Jordi Miquel, Samira Azzouz-Maache, Anne-Françoise Pétavy

**Affiliations:** 1 Witold Stefański Institute of Parasitology, Polish Academy of Sciences, 51/55 Twarda Street, 00-818 Warszawa, Poland; 2 Secció de Parasitologia, Departament de Biologia, Sanitat i Medi Ambient, Facultat de Farmàcia i Ciències de l’Alimentació, Universitat de Barcelona, Av. Joan XXIII, sn, 08028 Barcelona, Spain; 3 Institut de Recerca de la Biodiversitat (IRBio), Facultat de Biologia, Universitat de Barcelona, Av. Diagonal, 645, 08028 Barcelona, Spain; 4 Laboratoire de Parasitologie et Mycologie Médicale, Faculté de Pharmacie, Université Claude Bernard-Lyon 1, 8 Av. Rockefeller, 69373 Lyon Cedex 08, France

**Keywords:** *Echinococcus multilocularis*, Taeniid cestodes, Oncospheral penetration glands, Oncospheral nerve cells, Functional ultrastructure, Cytochemistry

## Abstract

The fine structure of the infective hexacanths of *Echinococcus multilocularis* was examined with particular emphasis on the functional ultrastructure of penetration glands and nerve cells directly involved in the mechanism of initial host infection. The oncosphere contains two types of penetration glands, PG1 and PG2, that differ slightly in size and form a large U-shaped bi-nucleated syncytial structure. The arms of each gland at each end of the U, directed towards the hook region, exit into the tegument peripheral layer between the median and lateral hook pairs. The lobate nuclei of PG1 and PG2 contain prominent spherical nucleoli surrounded by several large electron-dense islands of heterochromatin. The syncytial cytoplasm of both types of glands is rich in free ribosomes, polysomes, several mitochondria, and heavy accumulations of discoid secretory granules of moderate to high electron density. The secretory granules, sg1 and sg2, differ in their ultrastructure and electron density; the sg2 are much smaller and more flattened in shape. A common characteristic for sg1 and sg2, evident under high magnification, is their high electron density and discoidal shape, with two bi-concave surfaces. Both sg1 and sg2 are frequently grouped in characteristic parallel stacks, the “rouleau”-shaped assemblages with sometimes six to ten granules. Two nerve cells of neurosecretory type are situated in the central part of the hexacanth, each one in a deep U-shaped invagination between the two penetration glands. The nuclei of nerve cells contain several large heterochromatin islands closely adjacent to their nuclear membranes. Their cytoplasm is characterized by having membrane-bound, dense-cored neurosecretory-like granules not only in nerve cell perikarya but also in the elongated nerve processes frequently adjacent to gland arms and to both somatic or body musculature, including the complex system of hook muscles. The results of the present study, when supported with literature data on oncospheres of other cestode species, allow for a better understanding of the important role and coordinated functions of three structural components, i.e., oncospheral hooks, penetration glands, and nerve cells, in the mechanism of intermediate host infection. Presence or absence of nerve cells in oncospheres of various cestodes is reviewed, and perspectives on the value and application of research on functional morphology of oncospheres are discussed.

## Introduction

Both *Echinococcus granulosus* and *E. multilocularis* have been studied extensively owing to their medical importance and the economic losses they cause in some species of livestock. Of the two cestode species, however, much more is known of *E. granulosus* and the ultrastructural aspects of its reproductive and developmental biology (Świderski and Eckert 1977; Świderski 1982, 1983). Relatively little is known about the fine structure of mature, infective oncospheres of *E. multilocularis* and the three structural components, i.e., oncospheral hooks, penetration glands, and nerve cells, that are directly involved in the mechanism of intermediate host infection. The present study completes a two-part investigation, one on the morphogenesis and functional ultrastructure of oncospheral hooks of *E. multilocularis*, published recently (Świderski et al. 2016a), and the second part providing evidence for the existence of two types of penetration glands and the nerve cells within the infective larval form.

In cestode oncospheres, the complex hook and body movements observed during hexacanth activation, hatching, and host tissue penetration are evidence of coordinated activities that are likely mediated by some kind of neurotransmission. The presence of nerve cells in the oncosphere, however, was never reported in light microscope studies (for review, see Rybicka 1966), or in early electron microscopy papers (for reviews, see Świderski 1972, 1981, 1983).

In addition, ultrastructural analyses of infective cestode larvae are essential to our understanding of cestode biology and parasite and host interactions, especially the prevention of infection. Of particular interest is how information on the ultrastructure aspects of the oncosphere can be used in studies on immuno-localization of the host-protective antigens in the penetration glands of taeniid oncospheres (Jabbar et al. 2010a, b) and the possible development of new vaccines. In this regard, a number of practical and highly effective vaccines against other cestode infections, such as against hydatidosis and cysticercosis, have been developed and shown to be highly effective in preventing infection in the intermediate hosts (Lightowlers et al. 2000; Lightowlers 2006). It is significant that the most effective of these vaccines have utilized antigens obtained from the oncospheral stage (Lightowlers et al. 2003; Lightowlers 2006). A more recent review at the light microscope level with application immunocytochemistry methods is that of Hartenstein and Jones (2003).

Preliminary results on the ultrastructure of oncospheral nerve cells in *E. multilocularis* by Świderski (1994a) and *E. granulosus* and *E. multilocularis* by Świderski (1997) were first obtained about 12 years ago and published in abstract only. The purpose of the present study is a re-examination of the earlier studies and re-description of functional ultrastructure of the oncospheral nerve cells of *E. multilocularis*, with application of new, much improved modern methods of TEM involving freeze substitution with embedding in Lowicryl resin and TEM cytochemistry.

## Materials and methods

### Materials

Live specimens of *Echinococcus multilocularis* were isolated from the intestine of a naturally infected red fox (*Vulpes vulpes* L.) from La Roche sur Foron (France) captured in June 2014.

### TEM preparation of samples

Adult tapeworms were immediately rinsed with a 0.9% NaCl solution. Later, they were fixed in cold (4 °C) 2.5% glutaraldehyde in a 0.1 M sodium cacodylate buffer at pH 7.4 for a minimum of 2 h, rinsed in 0.1 M sodium cacodylate buffer at pH 7.4, post-fixed in cold (4 °C) 1% osmium tetroxide with 0.9% potassium ferricyanide in the same buffer for 1 h, rinsed in MilliQ water (Millipore Gradient A10), dehydrated in an ethanol series and propylene oxide, embedded in Spurr’s resin and polymerized at 60 °C for 72 h.

Ultrathin sections (60–90 nm thick) of gravid proglottides at different levels were obtained in a Reichert-Jung Ultracut E ultramicrotome. Sections were placed on 200-μm mesh copper grids and double-stained with uranyl acetate and lead citrate according to the Reynolds (1963) methodology. The grids were examined in a JEOL 1010 transmission electron microscope (Jeol, Japan) operated at 80 kV, in the “Centres Científics i Tecnològics” of the University of Barcelona (CCiTUB).

### Freeze substitution and infiltration with Lowicryl resin

Some specimens were fixed in cold (4 °C) 4% paraformaldehyde + 0.1% glutaraldehyde in 0.1 M sodium cacodylate buffer at pH 7.4 for a 4 to 5 h, and then conserved in cold (4 °C) 2% paraformaldehyde in the same buffer. Samples were rinsed in 0.15 M glycine in 0.1 M sodium cacodylate buffer at pH 7.4, cryoprotected by crescent concentrations (10, 20, and 30%) of glycerol in the same buffer, and then cryofixed in liquid propane.

Samples were freeze-substituted for 3 days at − 90 °C in anhydrous acetone containing 0.5% uranyl acetate. Then, they were warmed up to − 50 °C, at 5 °C/h (EM AFS2, Leica, Vienna, Austria). After several acetone rinses, samples were infiltrated with Lowicryl HM20 resin during 4 days. Samples were polymerized under UV light: at − 50 °C for 24 h, during the warming up at a rate 5 °C/h until 22 °C, and 48 h at 22 °C.

Ultrathin sections were picked up on Formvar-coated nickel grids, double-stained with uranyl acetate and lead citrate, and examined in a JEOL 1010 TEM operated at 80 kV, in the CCiTUB.

### Cytochemistry

The periodic acid-thiosemicarbazide-silver proteinate (PA-TSC-SP) technique of Thiéry (1967) was applied to determine the cytochemical localisation of glycogen at the ultrastructural level. Thus, ultrathin sections collected on gold grids were treated as follows: 30 min in 10% PA, rinsed in MilliQ water, 24 h in TCH, rinsed in acetic solutions, and MilliQ water, 30 min in 1% SP in the dark, and rinsed in MilliQ water. Gold grids were also examined in a JEOL 1010 TEM operated at an accelerating voltage of 80 kV, in the CCiTUB.

## Results

### Functional ultrastructure

**Fig. 1:**
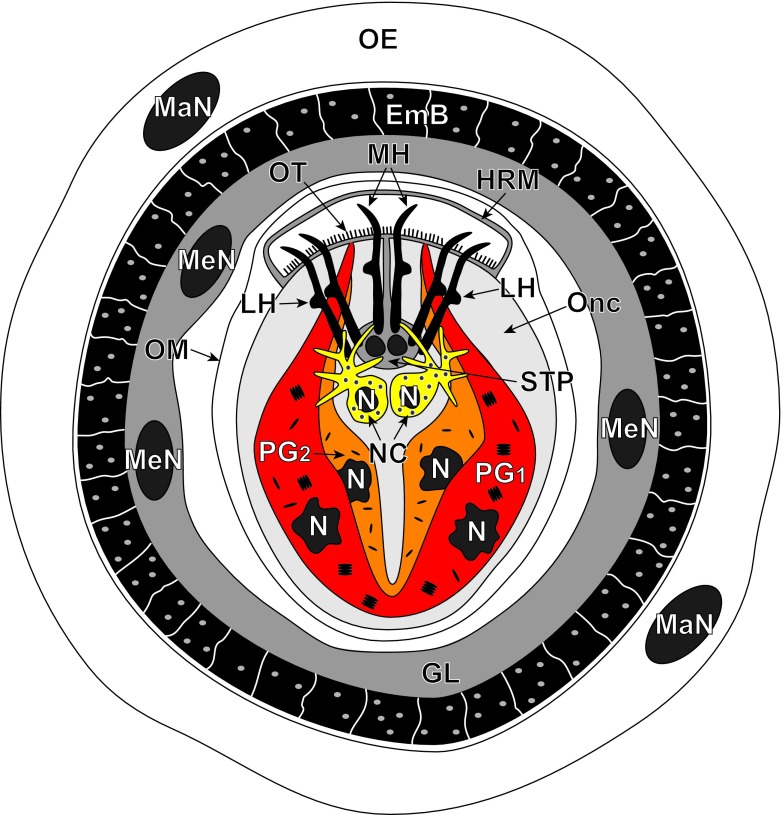
Schematic diagram illustrating localization of three secretory regions of the egg of *Echinococcus multilocularis*: two types of the penetration glands (PG1 and PG2) and two nerve cells (NC) of neurosecretory type. Note the oncospheral tegument composed of its peripheral anucleated layer and submerged subepithelial perikaryon and the hook region membrane surrounding the somatophore pole of the hexacanth. To simplify the diagram, some other oncospheral structures or cell types such as somatic and hook muscle systems with their perikarya or “somatic cells” and “germinative cells” have been purposely omitted. EmB embryophoric blocks of keratin-like protein, GL granular layer, HRM hook region membrane, LH lateral hooks, MaN two macromere nuclei of the outer egg envelope, MeN three mesomere nuclei of the inner egg envelope, MH median hooks, N nucleus, OE outer envelope, OM oncospheral membrane, Onc oncosphere, OT oncospheral tegument, STP bi-nucleated subtegumental perikaryon

The schematic diagram of mature egg of *E. multilocularis* (Fig. 1) shows the general topography and bilateral symmetry in cellular organization of the infective hexacanth larva surrounded by its four egg envelopes. The egg is usually ovoid in shape and measures about 30 μm in diameter. Three oncospheral structures or cell types described in this paper, nerve cells and two types of penetration glands, PG1 and PG2, are in three different colors for better visualization of their structure and the general topography of the hexacanth body. In order to focus on these key structures and simplify the scheme, other oncospheral structures or cell types such as somatic and hook muscle systems, “somatic cells” and “germinative cells” have been purposely omitted.

With regard to oncosphere terminology concerning its polarity or topographic orientation, we follow the terminology proposed by Ogren (1971) for the so-called primary or somatic polarity which occurs during differentiation stages and in infective oncospheres in distinction from the secondary or “germinative polarity” which determines the pattern of post-embryonic development of metacestode (see Mackiewicz 1984 for details and review of oncospheral polarity and its reorientation during cestode developmental stages). Such terms as “anterior pole” and “posterior pole” of the oncosphere are used in this paper with respect to hexacanth invasive activity. Larvae use hooks, generally in conjunction with penetration glands secretion, to penetrate through host tissue with the hooks oriented in the direction of movement. Therefore, the hook region, containing hook muscle system with exits of both types of penetration glands, directed forward during movement, is considered the anterior part of the larvae and functionally as the “somatophore.” The opposite hemisphere, containing penetration gland perikarya and germinative cells, is considered as posterior and functionally as “germatophore” or “mesophore.” The mature hexacanth is armed with three pairs of hooks, one medial pair and two lateral pairs interconnected by a complex hook muscle system (for details and review, see Świderski 1983; Świderski et al. 2016a). The exits of both types of the penetration glands are situated between the medial and lateral hooks (Fig. 1).

### Penetration glands

**Fig. 2:**
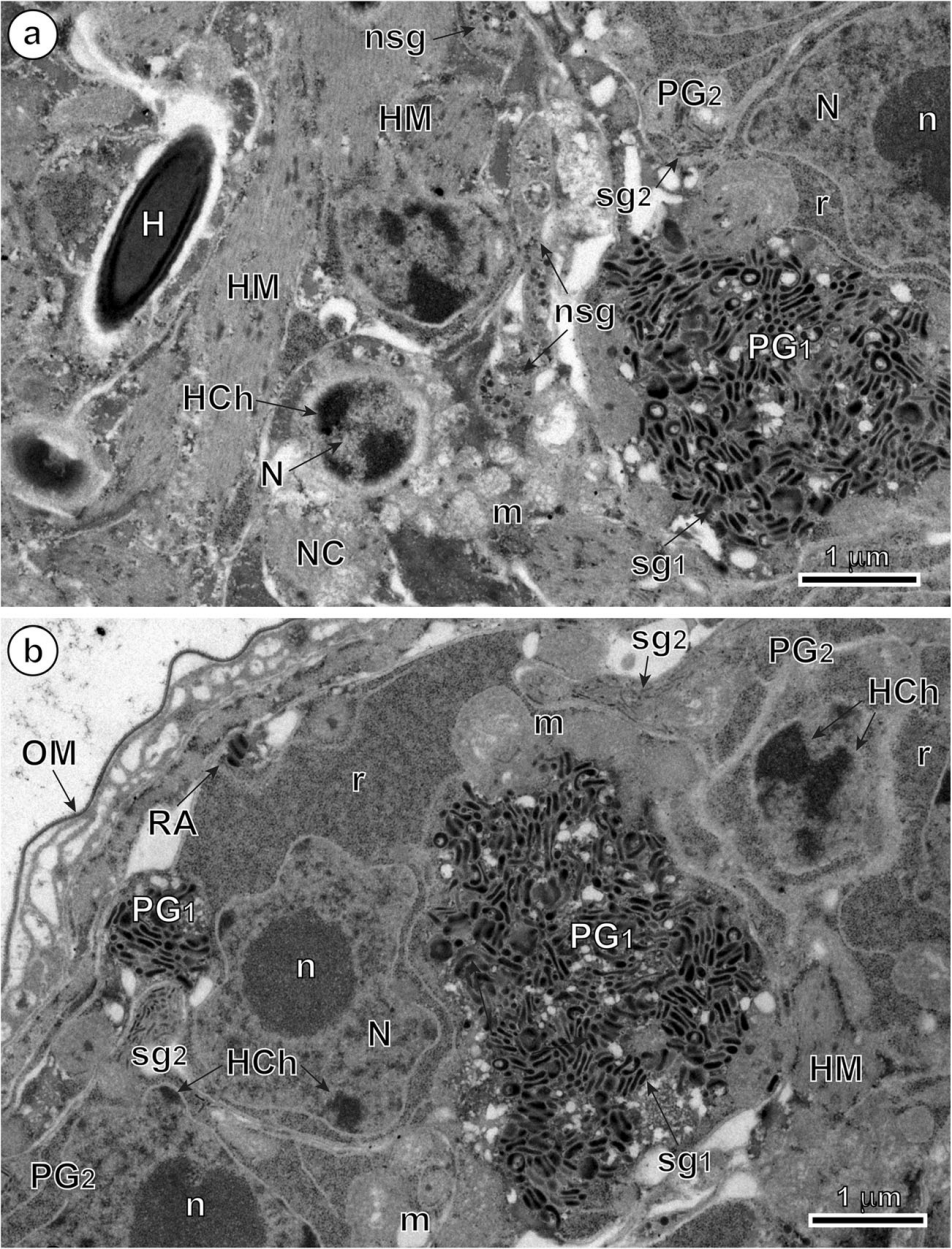
Details of two infective hexacanths of *Echinococcus multilocularis* obtained by means of freeze substitution technique. **a** TEM micrograph showing the two penetration glands (PG1 and PG2) and a nerve cell (NC). Note: (i) the nucleus (N) of a nerve cell with heterochromatin islands (HCh), (ii) the presence of numerous mitochondria (m) and (iii) the neurosecretory granules (nsg) located in a nerve cell process. **b** Area of hexacanth with two penetration glands (PG1 and PG2). Note the “rouleau”-shaped assemblages (RA) of secretory granules of the type 1 penetration gland (sg1). H oncospheral hook, HM hook musculature, n nucleolus, OM oncospheral membrane, r ribosomes, sg2 secretory granules of the type 2 penetration gland

**Fig. 3:**
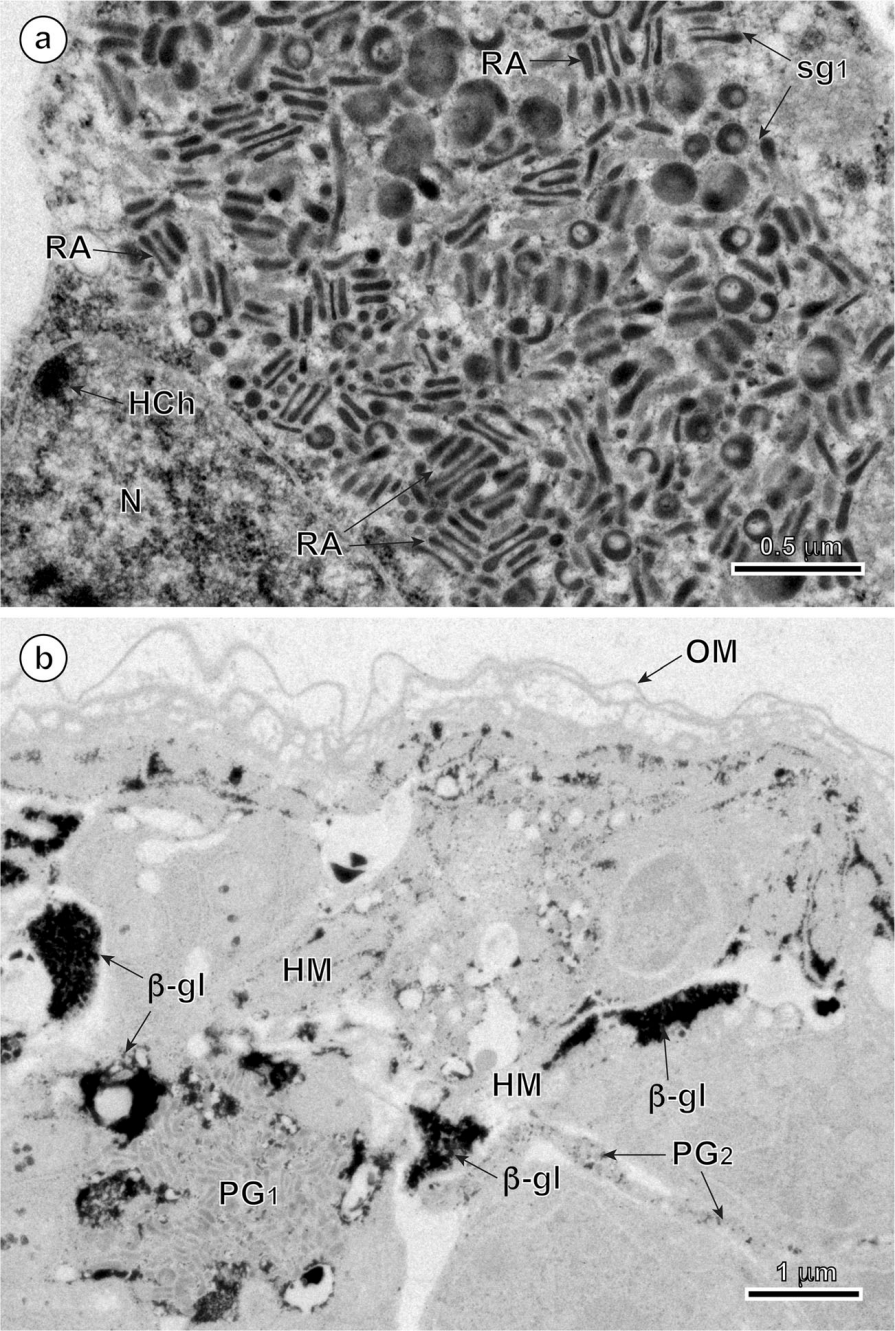
Enlarged details of the secretory granules sg1 in the type 1 penetration gland of *Echinococcus multilocularis* oncosphere and the cytochemical test of Thiéry for ultrastructural evidence of glycogen. **a** High power TEM micrograph showing numerous “rouleau”-shaped assemblages (RA) of the secretory granules of the type 1 penetration gland (sg1). **b** Cytochemical test of Thiéry showing the presence of large number of beta-glycogen particles (β-gl) around the two types of penetration glands (PG1 and PG2); results obtained after freeze substitution technique. HCh heterochromatin islands, HM hook musculature, N nucleus, OM oncospheral membrane

**Fig. 4:**
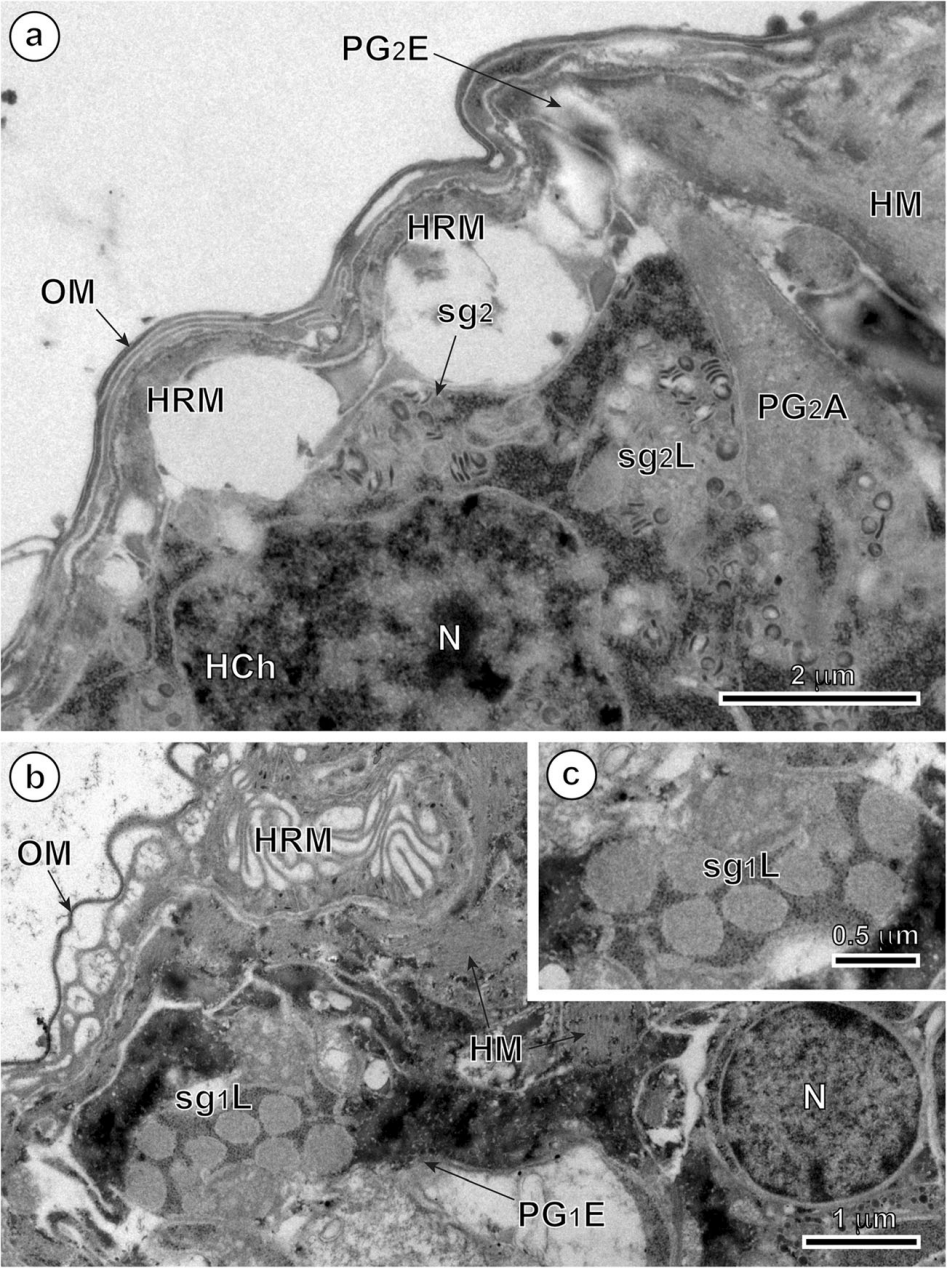
TEM micrographs of the higher magnification showing exit regions of the *Echinococcus multilocularis* penetration glands. **a** Part of the oncosphere showing the exit of the type 2 penetration gland (PG2E). Note the liquefied aspect of their secretory granules (sg2L). **b** Part of the hexacanth near the exit of the type 1 penetration gland (PG1E). Note the liquefied aspect of their secretory granules (sg1L). **c** Enlarged detail of the liquefied secretory granules of the type 1 penetration gland (sg1L). HCh heterochromatin islands, HM hook musculature, HRM hook region membrane, N nucleus, OM oncospheral membrane, PG2A arm of the type 2 penetration gland, sg2 secretory granules of the type 2 penetration gland

**Fig. 5:**
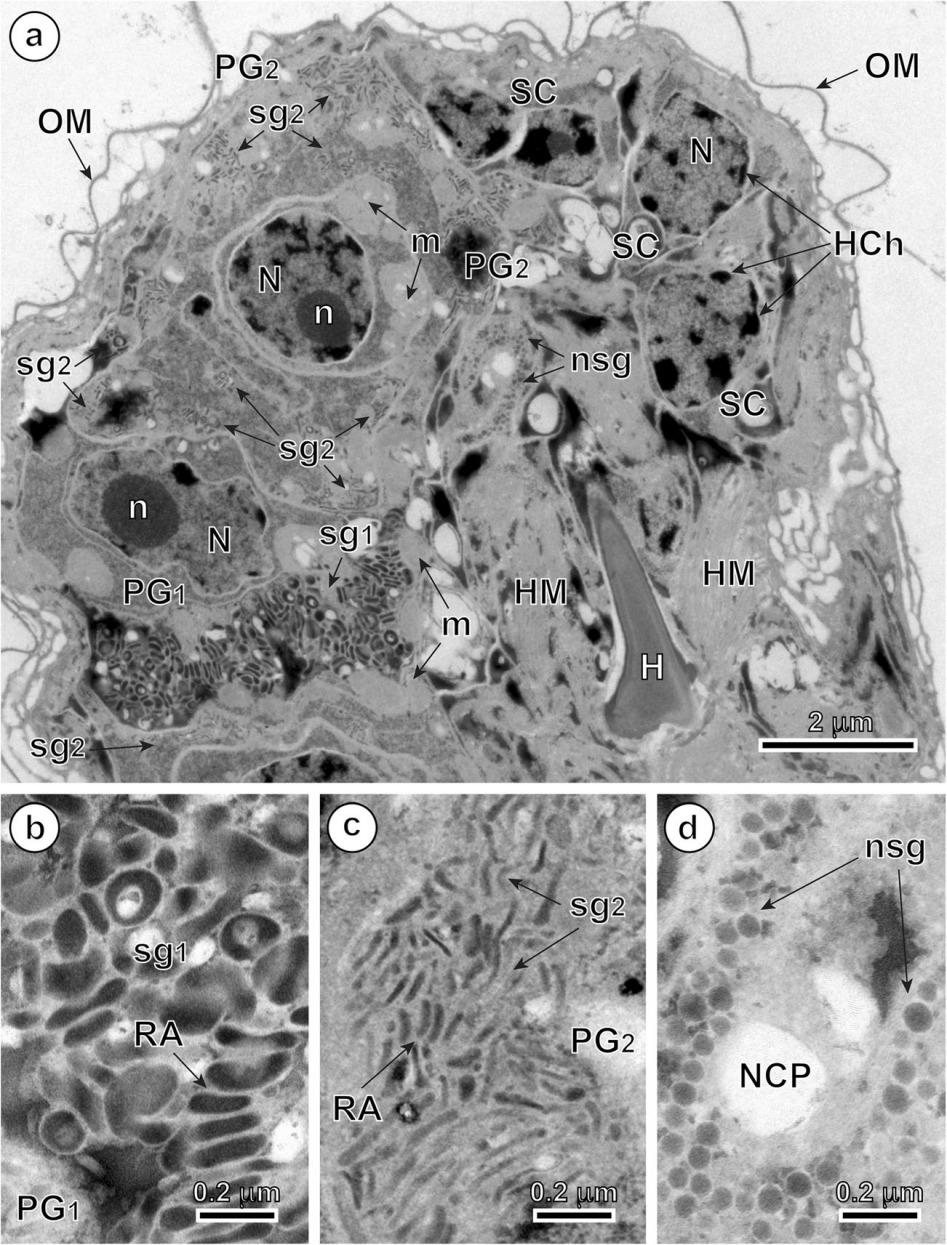
Oncospheral secretory regions of the hexacanth of *Echinococcus multilocularis* and comparison of three types of their secretory granules. **a** Somatophore area of hexacanth showing few secretory regions containing three types of secretory granules: sg1, sg2, and nsg granules. Two types of penetration glands, PG1 and PG2, show evidently different types of their secretory granules sg1 and sg2. **b–d** Higher magnification TEM micrographs showing ultrastructural details of three types secretory granules. sg1 (or sg2) represents secretory granules of the first (or second) type of penetration glands and nsg are neurosecretory granules of neurosecretory cells. H oncospheral hook, HCh heterochromatin islands, HM hook musculature, m mitochondria, N nucleus, n nucleolus, NCP nerve cell process, OM oncospheral membrane, RA “rouleau”-shaped assemblages of secretory granules, SC somatic cell

**Fig. 6:**
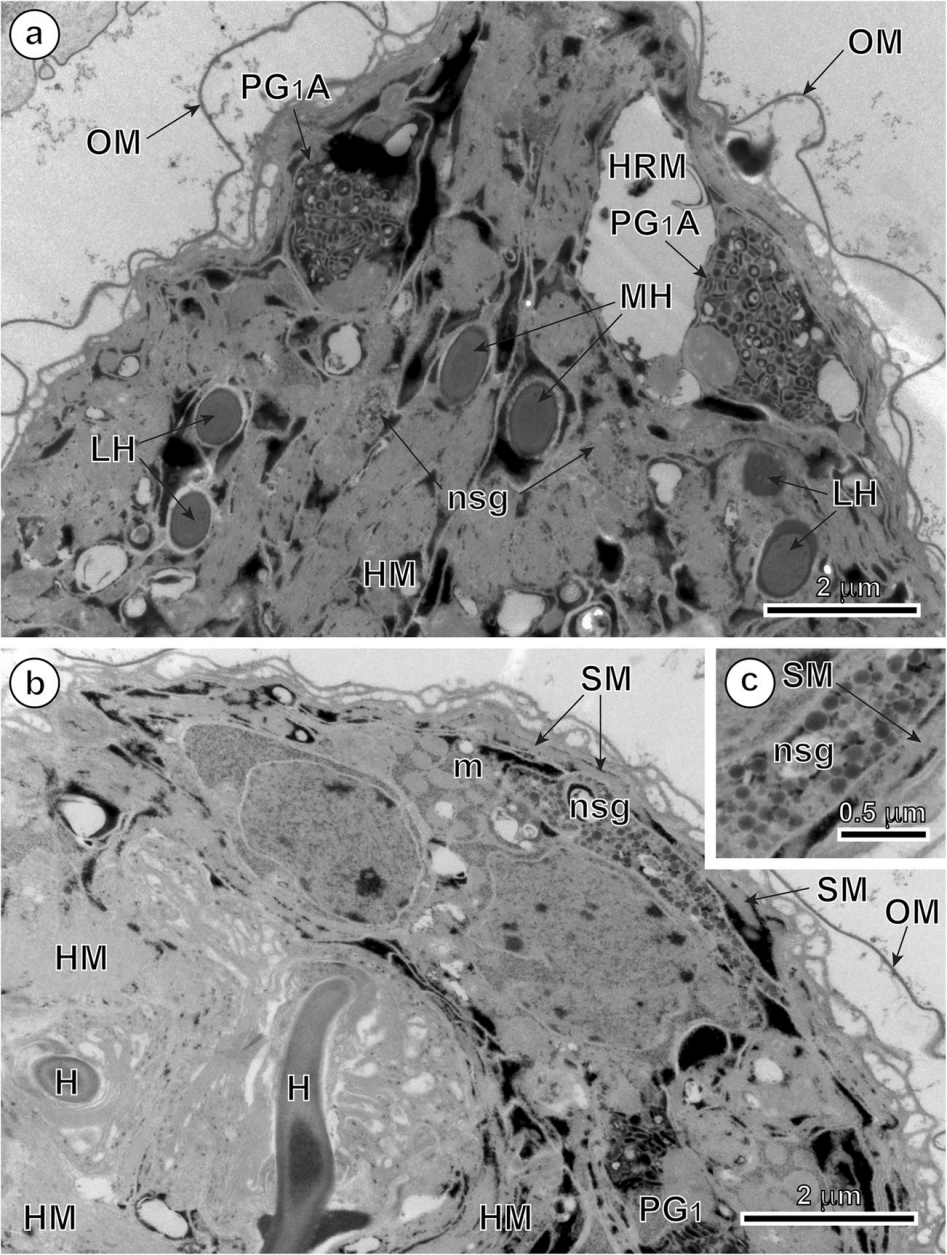
TEM micrographs of the anterior, somatophore region of the oncosphere of *Echinococcus multilocularis*. **a** Pole of the hexacanth showing a bilateral symmetry. Note the presence of the arms of the type 1 penetration gland (PG1A) between the median (MH) and lateral (LH) pairs of oncospheral hooks. **b** Detail of an oncosphere with the peripheral disposition of a nerve cell process containing neurosecretory granules (nsg). **c** Enlarged detail of neurosecretory granules (nsg). H oncospheral hook, HM hook musculature, HRM hook region membrane, m mitochondria, OM oncospheral membrane, PG1 type 1 penetration gland, SM somatic musculature

In mature, infective oncospheres of *E. multilocularis*, both gland types of the U-shaped penetration glands, PG1 and PG2, form two bi-nucleated syncytia with their two nuclei localized in the posterior, enlarged parts of each syncytial perikaryon of PG1 and PG2 (Fig. 1). Each type of gland has two arms, directed towards the hook region, which open into the tegumental peripheral layer as gland exits between the pairs of medial and lateral hooks (Fig. 1). The nuclei of both types of fully developed glands are lobate or amoeboid and contain in their karyoplasm prominent spherical nucleoli surrounded by several large islands of heterochromatin, all of high electron density (Figs. 2a, b, 4a, and 5a). The nuclei of both types of glands are surrounded by granular syncytial cytoplasm rich in free ribosomes, polysomes, several mitochondria, and heavy accumulations of discoid secretory granules of moderate to high electron density (Figs. 2a, b, 3a, and 5a). In both types of penetration glands, PG1 and PG2, their secretory granules sg1 and sg2, when observed under relatively low magnification appear essentially similar (Figs. 2a, b and 5a). Their characteristic differences are much more evident under high magnification (compare Fig. 5b, c). A common characteristic for both types of secretory granules sg1 and sg2, however, is their discoidal, flattened shape, with two concave surfaces, that are frequently grouped in characteristic “rouleau”-shaped assemblages sometimes six to ten granules (Figs. 3a, 5a–c, and 6a, b). The two types of secretory granules, sg1 and sg2, differ in their ultrastructure and electron density; the sg2 are much smaller and more flattened in shape. Most of the gland cytoplasm is filled with an accumulation of moderately electron-dense membrane-bound secretory granules, arranged in some parts in parallel stacks or “rouleau.”

In the arms of both types of penetration glands and particularly near the gland exits was observed liquefaction of both types of secretory granules (Fig. 4a–c). They represent probably the intermediate or transitional stages occurring during their maturation, liquefaction, and/or structural changes prior to secretion. These types may have, however, similar chemical composition.

### Nerve cells

Two nerve cells are situated in the central region of the oncosphere below the bases of oncospheral hooks, in the deep U-shaped invagination between two penetration glands PG1 and PG2 (Fig. 1). Their nuclei contain large osmiophilic heterochromatin islands usually adjacent to the nuclear membrane (Fig. 2a). Moreover, nerve cells are characterized by numerous membrane-bound, dense-cored neurosecretory-like granules in their cytoplasm (Figs. 2a, 5a, d, and 6b, c). In addition to their presence in the nerve cells perikarya, the typical neurosecretory granules were also frequently observed in the elongated nerve processes (Figs. 2a and 6a, b), frequently adjacent to the somatic or body musculature and to a complex system of the hook muscles (Fig. 6b, c).

### Cytochemistry

Cytochemical parts of our study were made on the material processed by means of a freeze substitution method and embedded with Lowicryl resin; its results are illustrated on Fig. 3b. The results of the test of Thiéry demonstrate the presence of large numbers of individual beta-glycogen particles (β-gl) dispersed around the peripheral cytoplasmic layers in the two types of penetration glands (PG1 and PG2). Positive and frequently much stronger positive reaction for beta-glycogen was also observed in the adjacent somatic and hook musculature of the oncosphere (Fig. 3b).

## Discussion

### Penetration glands

The term “penetration glands” was introduced by Reid (1948), who suggested that the secretion of these oncospheral glands may help hexacanths penetrate the tissues of the intermediate host. There has been some question in the past about the presence of penetration glands in the coracidium. In her review on cestode embryogenesis, Rybicka (1966, p. 165) wrote, “...excretory glands, in form of unicellular protonephridia, have been described only in pseudophyllidean oncospheres, whereas in cyclophyllidean oncospheres only secretory penetration glands have been found so far. This difference is surprising, particularly when we consider the general similarity of oncospheres in both groups and also their behaviour in the intermediate host.” And she continues “...we do not understand why coracidia lack penetration glands when such glands are well developed in cyclophyllidean oncospheres which infect the same intermediate hosts.”. It was not until coracidia had been studied at the ultrastructural level that this apparent paradox could be resolved. As Kuperman (1988) demonstrated, both the penetration glands and flame cells are present in coracidia of *Bothriocephalus acheilognathi*, *Triaenophorus crassus*, and *Diphyllobothrium latum*. More recently, it was also shown by Świderski and Mackiewicz (2004) that the coracidia of *Bothriocephalus clavibothrium* have both penetration glands and flame cells or excretory glands, according to Rybicka’s terminology.

Initially, the penetration glands have been described as comprising two cells located symmetrically behind the hooks and joined together by an isthmus to give a U-shaped syncytial structure. The nuclei lie at the base of the U and the cytoplasm contains numerous secretory granules (Reid 1948). Later, in some light and electron microscopy studies of cestode oncospheres, the penetration glands were described as bi-nucleated, four-nucleated, multicellular or unicellular glands (for review, see Świderski and Tkach 2002). The most common among different cestode species is, however, the U-shaped syncytial bi-nucleated type of penetration gland.

The mechanism of penetration gland secretion has been classified as merocrine, apocrine, or holocrine (for reviews see Lethbridge and Gijsbers 1974; Lethbridge 1980; Świderski and Tkach 1997; Młocicki et al. 2006, 2010). In spite of the above hypotheses, the mechanism of secretion from the penetration glands still remains unclear because there is no direct evidence of cell contents being secreted.

Secretory materials produced by the penetration glands appear heterogeneous ultrastructurally (Lethbridge and Gijsbers 1974), are periodate-reactive (Lethbridge 1971), and absorb the cationic dye neutral red (Lethbridge and Gijsbers 1974). These data indicate the presence of acidic carbohydrate-rich mucosubstance and potentially proteolytic enzymes. A number of descriptions of the secretory products characterize them as including: (1) a proteinaceous substance with enzymatic properties (Pence 1970), e.g., serine proteinase (Moczoń 1996); (2) an acid mucoprotein (Fairweather and Threadgold 1981); (3) an acidic mucopolysaccharide (Heath 1971; Świderski and Eckert 1977; Świderski 1994b); and (4) a polysaccharide (Silverman and Maneely 1955 and the results of the present study).

### Immunological role of penetration glands

A possible immunological role of the oncospheral penetration glands has been described and discussed by Silverman and Maneely (1955) who initially suggested and speculated on the potential antigenic nature of penetration gland secretions, but there has been no direct evidence to support this hypothesis. The proposition is supported indirectly by evidence that, during the first few hours of host invasion, the glands constitute a large volume as compared with the total size of the oncosphere, and they secrete macroscopic material (Silverman and Maneely 1955). For an extensive review of host-protective antigens and oncospheres see Jabbar et al. (2010a, b).

### Nerve cells

The presence of nerve cells in cestode oncospheres was never reported in light microscopical studies (for review, see Rybicka 1966; Ogren 1968a, b), and initially, it was believed that they are absent in the hexacanth larvae. Rybicka (1967) demonstrated acetylcholinesterase activity in mature oncospheres of *Hymenolepis diminuta* but was unable to find a specific nerve cell or nerve centre. Since the oncospheral muscular system is quite extensive, she suggested that the presence of acetylcholinesterase activity is not surprising, if it is indeed associated with myofibres. It should be remembered that in all cestode oncospheres so far examined, only a single type of dense-cored vesicle was detected; consequently, it can be presumed that acetylcholine does not function as a neurotransmitter in hexacanth larvae. Nerve cells in an oncosphere were first described in the hexacanths of *Hymenolepis nana* by Fairweather and Threadgold (1981). Hartenstein and Jones (2003) have presented by means of light microscopy (LM) and LM immunocytochemistry clear details of the presence of nerve cells also in the mature oncosphere of *Hymenolepis diminuta*. Their presence was also demonstrated in oncospheres of *Echinococcus granulosus* (Świderski 1982, 1997) and *E. multilocularis* (Świderski 1994b, 1997), but preliminary results were published only in short congress abstracts. The results of our present study confirm entirely these preliminary data. That nerve cells are present in a variety of other cestode oncospheres as demonstrated in several other TEM studies describing ultrastructure of oncospheral cells in a few other cestode species, e.g., *Ditestolepis tripartita* (see Świderski and Tkach 1997), *Pseudhymenolepis redonica* (see Tkach and Świderski 1997), *Inermicapsifer madagascariensis* (see Świderski and Tkach 2002), *Mosgovoyia ctenoides* (see Młocicki et al. 2005, 2006), and *Eubothrium salvelini* (see Młocicki et al. 2010). The data on the above cestode species show the presence of neurosecretory granules in elongated nerve cell processes situated near the hook and somatic musculature as well as near the penetration glands, which may confirm their important function in coordination of hook and body movements and secretory activity of the penetration glands. One potential disagreement with earlier literature is whether there are in some species more than one type of dense-cored vesicle in neurons of some species of lower cestodes and amphilinids (see Korneva 2001, 2004; Biserova and Korneva 2006). Our results with *E. multilocularis* show only one type of dense-cored vesicles in the oncospheral nerve cells as was also demonstrated in the abovementioned cestodes (see Tkach and Świderski 1997; Świderski and Tkach 1997, 2002; Świderski and Mackiewicz (2004; Młocicki et al. 2005, 2006, 2010). Among these species are not only cyclophyllideans or higher cestodes but also two species of lower cestodes, namely *Bothriocephalus clavibothrium* (see Świderski and Mackiewicz 2004) and *Eubothrium salvelini* (see Młocicki et al. 2010). On the other hand, nerve cells were absent from oncospheres of *Catenotaenia pusilla* according to Świderski (1972) and *Hepatocestus hepaticus* according to Świderski et al. (2000), both with an evident or presumed passive mode of transmission to their intermediate hosts. The passive mode, in case of *C. pusilla*, was confirmed experimentally by Joyeux and Baer (1945) and in case of *H. hepaticus* was presumed and discussed by Świderski et al. (2000). To what extent the presence or absence of nerve cells in the oncospheres may be related to an active or passive mode of transmission to intermediate hosts has yet to be determined. Classification of those cells into a neurosecretory type, as initially emphasized by Fairweather and Threadgold (1981), was based on purely cytological criteria. It is clear, however, that since nerve cells are apparently not present in all cestode oncospheres, additional information is needed to confirm this viewpoint and to better understand neurosecretory function (Świderski et al. 2001).

### Cytochemical results

As described in the “Results” section, the cytochemical technique of Thiéry for glycogen localization at the ultrastructural level shows a strongly positive reaction for beta-glycogen in both types of oncospheral musculature, namely peripheral somatic muscles and in a specialized system of hook muscles, as also demonstrated previously by Świderski et al. (2016a, 2018). This glycogen represents an important source of energy for a very active, synchronized action of oncospheral hooks and for rapid movements of the entire hexacanth during host tissue penetration. Similar results were obtained previously in our studies on hexacanths of other cestode species (Świderski and Mackiewicz 2004; Świderski et al. 2016b). The amount of beta-glycogen in the peripheral layer of cytoplasm of penetration glands PG1 and PG2 of *E. multilocularis* oncospheres is very limited, but comparable to that described in hexacanths of other cestode species (Świderski and Mackiewicz 2004; Świderski et al. 2016b). It certainly reflects the limited role of this substance as energy source for functioning of penetration glands of hexacanths.

### Conclusion and perspectives on practical application of results

Comparative results on glycogen cytochemistry in the penetration glands PG1 and PG2 and in the somatic and hook musculature of *E. multilocularis* oncospheres show positive reaction for beta-glycogen in both abovementioned structures, the rosettes of alpha-glycogen, usually serving for a long storage, were never observed. The beta-glycogen, considered as an important energy source for rapid in situ utilization is very abundant in somatic and hook musculature, but its amount in the penetration glands is very limited. Functionally, it reflects different roles of both types of oncospheral structures in the nechanism of intermediate host invasion and in the hexacant’s activity.

The aims in studying the functional ultrastructure of the penetration glands and nerve cells within the oncospheres of the taeniid cestode *E. multilocularis*, a parasite of man and animals, are twofold; one is for scientific interest in gaining better knowledge of the production of infective eggs of this unique cestode, what concerns its pathogenicity. It is well known that frequently results of some purely scientific research may open a new horizon for direct practical application in the future. The second aim concerns the parasitological and medical aspect that ultimately seeks a means of preventing infection. This ultimate goal might seek to alter the parasite cycle to having production of nonviable eggs, or by controlling or eradicating the parasite by interrupting its life cycle.

In targeting the neurosecretory activity of oncospheral nerve cells that control the penetration glands, enzymatic secretion and movements of oncospheral hooks one may be able to alter or prevent the infection process. The hope lies in finding new chemotherapeutic agents that can act specifically on some neuronal signals or receptors of the infective oncosphere, thus preventing its activation, hatching and ability to infect an intermediate host—thus achieving the ultimate goal of this parasitological research.
